# Investigation of Variation in Gene Expression Profiling of Human Blood by Extended Principle Component Analysis

**DOI:** 10.1371/journal.pone.0026905

**Published:** 2011-10-27

**Authors:** Qinghua Xu, Shujuan Ni, Fei Wu, Fang Liu, Xun Ye, Bruno Mougin, Xia Meng, Xiang Du

**Affiliations:** 1 Department of Pathology, Fudan University Shanghai Cancer Center, Shanghai, People's Republic of China; 2 Department of Oncology, Shanghai Medical College, Fudan University, Shanghai, People's Republic of China; 3 Fudan University Shanghai Cancer Center – Institut Mérieux Laboratory, Fudan University Shanghai Cancer Center, Shanghai, People's Republic of China; 4 bioMérieux, Centre Christophe Mérieux, Grenoble, France; 5 Institutes of Biomedical Sciences, Fudan University, Shanghai, People's Republic of China; University of Bristol, United Kingdom

## Abstract

**Background:**

Human peripheral blood is a promising material for biomedical research. However, various kinds of biological and technological factors result in a large degree of variation in blood gene expression profiles.

**Methodology/Principal Findings:**

Human peripheral blood samples were drawn from healthy volunteers and analysed using the Human Genome U133Plus2 Microarray. We applied a novel approach using the Principle Component Analysis and *Eigen-R* ^2^ methods to dissect the overall variation of blood gene expression profiles with respect to the interested biological and technological factors. The results indicated that the predominating sources of the variation could be traced to the individual heterogeneity of the relative proportions of different blood cell types (leukocyte subsets and erythrocytes). The physiological factors like age, gender and BMI were demonstrated to be associated with 5.3% to 9.2% of the total variation in the blood gene expression profiles. We investigated the gene expression profiles of samples from the same donors but with different levels of RNA quality. Although the proportion of variation associated to the RNA Integrity Number was mild (2.1%), the significant impact of RNA quality on the expression of individual genes was observed.

**Conclusions:**

By characterizing the major sources of variation in blood gene expression profiles, such variability can be minimized by modifications to study designs. Increasing sample size, balancing confounding factors between study groups, using rigorous selection criteria for sample quality, and well controlled experimental processes will significantly improve the accuracy and reproducibility of blood transcriptome study.

## Introduction

Peripheral blood is a very promising material for biomedical research due to its critical role in immune responses and metabolism. The ease and minimal invasiveness with which it can be collected have also made peripheral blood attractive for clinical use. Over the last decade, advances in microarray have offered the opportunity to study the expression of thousands of genes simultaneously in a biological system. The microarray-based transcriptome analyse of peripheral blood may provide new insights into the variations in global gene expression specifically associated with physiological and pathological events. Numerous studies have addressed the use of gene expression profiling of peripheral blood from patients with malignancies, infectious diseases, autoimmunity and cardiovascular diseases [1], [2].

Before the blood transcriptomic biomarkers can be used for clinical purposes, it is essential to understand the underlying factors that contribute to the sources of variability in blood gene expression profiles. Previous studies have investigated the blood gene expression profiles of normal individuals [3]. Those studies used either whole blood or peripheral blood mononuclear cells. Gene expression profiles were found varying greatly among the different blood cell types [4]–[6]. The results demonstrated the significant contributions of genetic and physiological factors, as well as varying proporations of different cellsubsets, in determining the overall gene expression profiles of human blood [7]–[9]. Yet other research also provided evidences that gene expression profiles of normal individuals can be remarkably stable over time [10], [11]. Several studies reported an excess of hemoglobin mRNA present in total RNA extracted from whole blood resulting in high noise and reduced sensitivity in transcriptome analysis [12], [13]. The usefulness of available methods to minimize excess hemoglobin mRNA was then evaluated [14]. Furthermore, technological factors such as sample collection, transportation and storage conditions, as well as RNA isolation and amplification techniques, in addition to biological factors, can have a significant impact on the blood gene expression profiles [15]–[20].

In the study described here, we used the PAXgene^TM^ Blood RNA System and GeneChip® U133Plus2 Microarray to analyze gene expression profiles in peripheral blood from healthy Chinese volunteers. For each donor, the physiological variables and blood cell counts were measured. The blood gene expression profiles were investigated for possible interference of age, gender, body mass index (BMI), sample RNA quality, as well as the presence of varying proportions of different blood cell types.

## Methods

### Human Blood Sample Collection

For gene expression profiling, 2.5 ml of peripheral blood were drawn from each volunteer by PAXgene^TM^ Blood RNA tubes (PreAnalytix, Hilden, Germany). Another 2 ml of blood were collected for Complete Blood Count analysis. The test was performed by standard procedures at the Fudan University Shanghai Cancer Center Clinical Laboratory. The measures included white blood cell counts (leukocyte counts, relative counts for neutrophils, lymphocytes and monocytes), red blood cell counts (erythrocyte counts, hemoglobin amount, and relative reticulocyte count), as well as platelet counts. This study was carried out at the Fudan University Shanghai Cancer Center and was approved by the Ethical Committee of Fudan University Shanghai Cancer Center for Clinical Research. The written informed consents were obtained from all participants.

### RNA Isolation and Preparation

**Figure 1:**
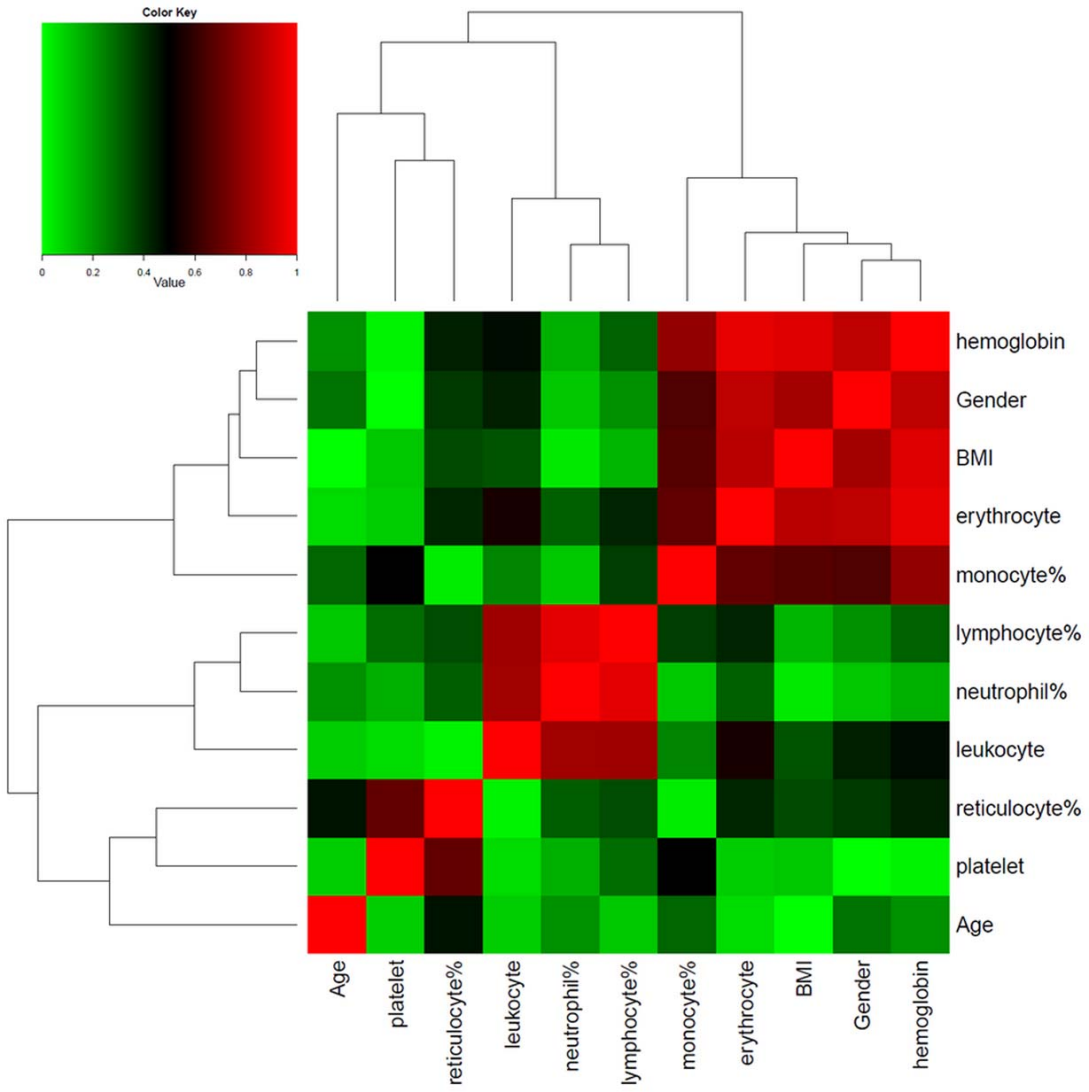
The study design for evaluating the impact of RNA quality on the blood gene expression profiles. From each healthy volunteer, 2.5 ml of peripheral blood was collected using a PAXgene^TM^ Blood RNA tube. The extracted total RNA from each individual was aliquoted into three tubes. The 1^st^ tube was kept intact, while the 2^nd^ and 3^rd^ tubes were heated at 70°C for 10 and 20 minutes, respectively. According to the RNAs processing, microarray experiments were then performed in the sequential series: Series_1, Series_2 and Series_3. The RIN value was measured and used as the technical variable for evaluating the impact of RNA quality on the blood gene expression profiles.

Once blood samples were drawn into the PAXgene^TM^ Blood RNA tubes by standard phlebotomy procedure, the samples were inverted ten times, maintained at room temperature for 2 hours, frozen at −20°C overnight and then moved to −80°C for storage until further use. Frozen blood samples were thawed at room temperature for 3 hours, followed by total RNA extraction with the PAXgene^TM^ Blood RNA kit according to the manufacturer's instruction. The intact total RNA of each participant was separated into three tubes (Figure 1). While the 1^st^ tube was kept intact, the 2^nd^ and 3^rd^ tubes were heated at 70°C for 10 and 20 minutes, respectively. The quantity of the total RNA was measured by a spectrophotometer at an optical density (OD) of 260 nm. Total RNA purity was assessed by the A260/A280 ratio. RNA Integrity Number (RIN) was determined using RNA 6000 Nano Chips and the Agilent 2100 Bioanalyzer (Agilent Technologies, Palo Alto, CA, U.S.A.).

### Microarray Experiments

We reversely transcribed 50 ng of total RNA and linearly amplified single-stranded cDNA using Ribo-SPIA™ technology with the WT-Ovation™ RNA Amplification System (NuGEN Technologies Inc., San Carlos, CA, USA) according to the manufacturer's standard protocol. The reaction products were purified with a QIAquick PCR purification kit (QIAGEN GmbH, Hilden, Germany). We subsequently fragmented 2 µg of amplified and purified cDNA with RQ1 RNase-Free DNase (Promega Corp., Fitchburg, WI, USA) and labelled the fragments with biotinylated deoxynucleoside triphosphates using terminal transferase (Roche Diagnostics Corp., Indianapolis, IN, USA) and GeneChip® DNA Labeling Reagent (Affymetrix Inc., Santa Clara, CA, USA). The labelled cDNA was then hybridized onto a GeneChip® U133Plus2 Array (Affymetrix) in a Hybridization Oven 640 (Agilent Technologies) at 60 rotations per minute and 50°C for 18 hours. After hybridization, the arrays were washed and stained according to Affymetrix protocol EukGE-WS2v4 using a GeneChip® Fluidics Station 450 (Affymetrix). The arrays were scanned with a GeneChip® Scanner 3000 (Affymetrix). All reactions and array hybridizations were carried out by the same technician to minimize the technical variation.

### Statistical Analysis

Statistical analysis was performed using R software and packages from the Bioconductor microarray analysis environment [21], [22], adapted to our needs. Gene expression profiles were quantified using the Robust Multi-array Average (RMA) method [23]–[25] implemented in the “Simpleaffy” package [26]. The GeneChip® U133plus2.0 Array contains 54,000 probesets for the interrogation of 38,500 human genes. Very often, multiple probesets are targeting to the unique gene. It is also found that several probesets were poorly annotated without any target gene. To reduce the noise and redundancy, we applied a bioinformatics-based filter using the information of Entrez Gene Database. For multiple probesets mapping to the same Entrez Gene ID, only the probeset showing the largest Inter Quantile Range (IQR) were retained and the others were removed. The probesets without Entrez Gene ID annotation were also removed. After bioinformatics-based filtering, the expression profiles of 9859 genes in 24 samples were retained for the downstream analysis.

Let 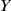 be a 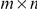
matrix, where the rows of 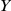 are the genes (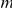 = 9859) and the columns of 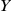 are the samples (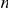 = 24). Although with the bioinformatics filtering, the difficulty remained in the fact that the number of genes was still much larger than the number of samples. Thus, we performed Principle Component Analysis (PCA) to reduce the dimensionality of gene expression data. In PCA, singular value decomposition was applied to the mean centered data matrix and decomposed 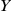 into the following: 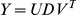



where the matrices 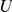 and 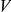
were column orthogonal, so that 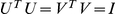 and 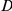 was a diagonal matrix. The columns of 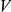
were the right eigenvectors and also called principle components (PCs). We were particularly interested in the PCs because these represented the aggregated trends in the gene expression profiles. Specifically, the first PC was the linear combination of the gene expression profiles that explained the most variation in the data. The second PC was the linear combination of the gene expression profiles that explained the most variation in the data once the first PC had been removed, and so on. The proportion of total variation captured by the 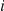-th PC was given by:
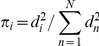



where 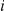 = 1,2,..,n; 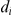 was the eigenvalue of the 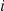-th eigenvector, which was obtained from the 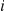-th diagonal entry of 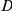. Afterwards, a PCA extended method, called “*Eigen-R^2^*” [27], was used to precisely determine the proportion of variation explained by the predefined biological and technological variables 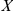 based on the PCA transformed data. Let 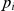 be the 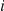-th PC and let 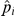 be the fitted values when modelling 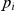 in terms of the predefined variable 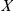. For each of PCs, the proportion of variation in 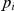 that is explained by 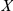 was calculated by:
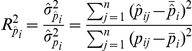



where 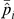 was the mean of 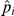 and 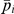 was the mean of 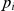. Since 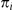 of the total variation in the data is explained by 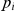, 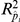 should be weighted by 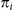. Additionally, given each pair of PCs is uncorrelated, the variation explained by 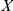 in 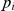 is orthogonal to the variation explained by 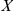 in 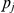 where 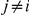. Therefore, the proportion of total variation explained by 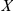 was estimated by:
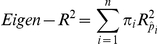



Given the small cohort of present study, we further adjusted the *R* ^2^ calculation to accommodate small sample sizes by:
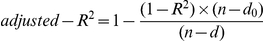



where 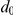 and 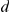 were the degrees of freedom spent in fitting the null model and the target model, respectively. By default, the null model was a model with only intercept (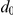 = 1). The adjusted estimator had been shown to be a unbiased estimator of the true value [28].

The gene-wise linear analysis was performed using the *lmFit()* function from the “Limma” package [29], adjusted for multiple testing with False Discovery Rate (FDR) [30]. For each gene, a linear model was fitted over the series of arrays with the log2-transformed gene expression intensity as the dependent variable and the biological and technological factors as the independent variables. The functional annotation enrichment analysis for gene ontology and pathways were performed by the DAVID Bioinformatics Resource (http://david.abcc.ncifcrf.gov) with default parameters [31].

The microarray dataset discussed in this study were MIAME compliant [32]. The raw data had been deposited in ArrayExpress Database [33] and were accessible through the accession number: E-MEXP-2917.

### Using the Independent Dataset for *in silico* Validations

Given the small sample size in the present study, we further used an independent, previously published blood gene expression dataset to verify our findings [10]. The Karlovich *et al.* study employed the same methodologies for the sample collection (PAXgene^TM^ Blood RNA System) and gene expression profiling (HG-U133Plus2 Microarray), hence we considered the dataset an appropriate material to perform the *in silico* validation. From the Karlovich *et al.* dataset, we selected a total of 100 arrays with blood samples drawn from 20 (11 female and 9 male) healthy volunteers at five time points (Starting day, Day 14, Day28, Day90 and Day 180). All volunteers were Caucasians and lived in eastern France. The volunteers ranged in age from 23 to 64 yr (mean ± SD = 46±15). The age distribution was well balanced between men and women. The raw data were downloaded from NCBI's Gene Expression Omnibus [34] with the accession number: GSE16028.

### Gene Expression Analysis by Real-time PCR

For each sample, 0.2 µg of total RNA was reverse-transcribed into cDNA using the *Prime Script* ^TM^ reverse transcriptase (TaKaRa, Dalian, China). Real-time PCR analysis was performed by the LightCycler® 480 system (Roche Diagnostics, Mannheim, Germany) in 96-well plates using the SYBR *Premix Ex Taq* ^TM^ (TaKaRa, Dalian, China) according to the manufacturer's instructions. Primer designs were provided in the Table S1. *ACTB* (β-actin) was used as an internal control. The relative quantification of mRNA expression was calculated as a ratio of target gene to *ACTB*. The correlations of real-time PCR data with microarray data and predefined variables were assessed by the Spearman's Rank Correlation Test.

## Results

### Sample Characteristics and Variable Definition

Peripheral blood samples were taken from 8 (4 female and 4 male) apparently healthy volunteers. All volunteers were Chinese, not on medication and non-fasted. The volunteers ranged in age from 22 to 35 yr (mean ± SD = 27.1±4.1). The age distribution was balanced between men and women. The volunteers' height and weight were measured and converted into BMI. The BMI ranged from 17.6 to 29.4 (mean ± SD = 21.3±3.7). For each volunteer, the blood cell counts including leukocyte count, lymphocyte%, monocyte%, neutrophil%, erythrocyte count, hemoglobin amount, reticulocyte% and platelet count were measured.

**Figure 2:**
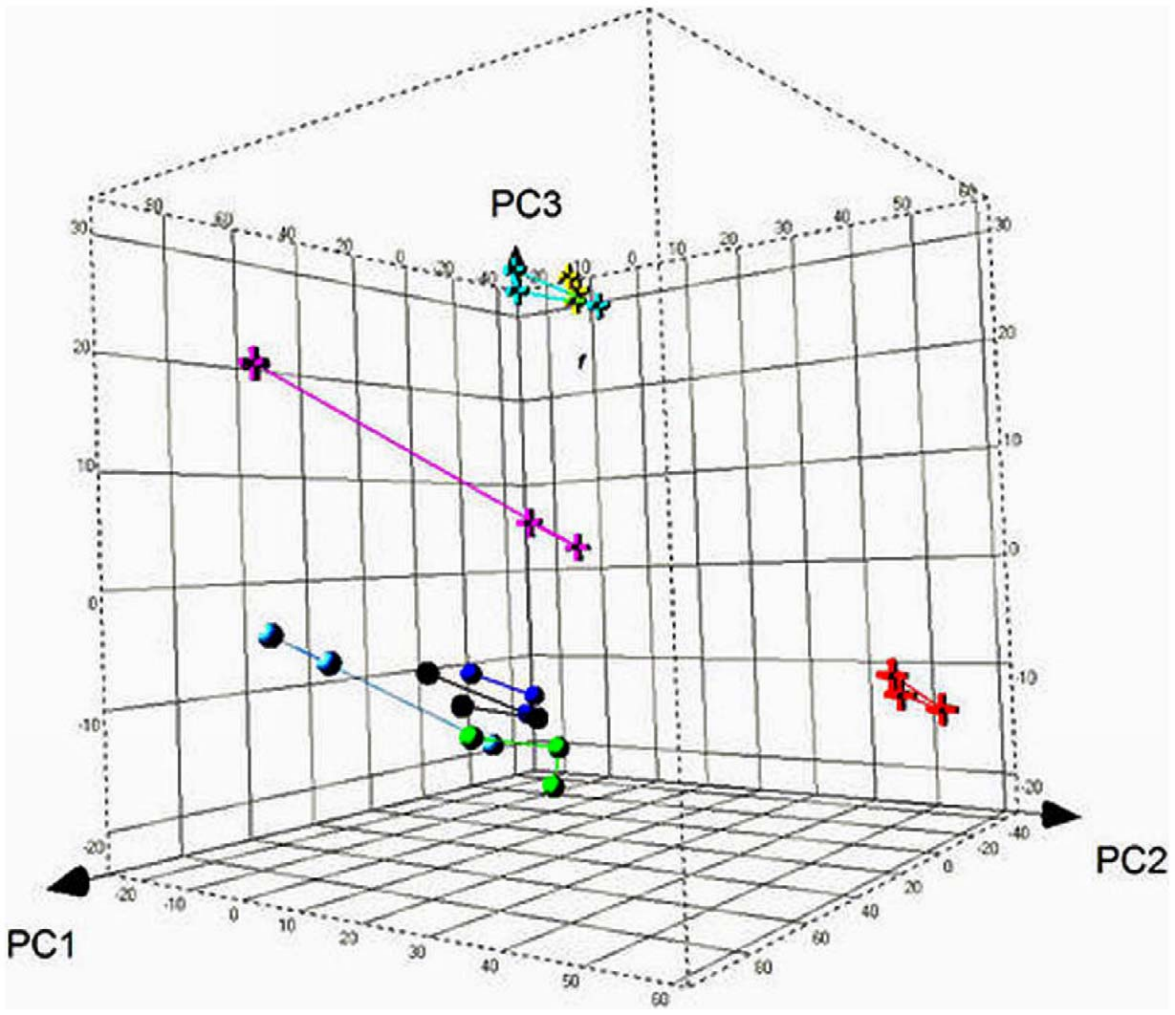
Heatmap of correlationship between demography variables and blood cell count measures. The Spearman's Rank Correlation Test was performed to assess the correlationship between age, gender, BMI and blood cell counts. The absolute values of correlation coefficients were represented as the heatmap with red colour indicating high correlations and green colour indicating low correlations between the variables.

We performed Spearman's Rank Correlation Test to explore the correlationship between age, gender, BMI and blood cell counts (Figure 2). Given that lymphocytes and neutrophils together making up 80–95% of total leukocytes, not surprisingly, the lymphocyte% and neutrophil% was inversely correlated with each other (ρ = −0.95, *P*-value<0.001). The erythrocyte count, hemoglobin amount, monocyte% as well as BMI were observed significantly correlated with the gender factor (*P*-value<0.001). The reticulocyte% and platelet count were also found to be correlated with each other (ρ = 0.69, *P*-value<0.001). The age variable was not apparently correlated with any other variable. Furthermore, the RIN value was measured to assess the RNA integrity on a scale from 0 (low integrity) to 10 (high integrity) [35]. Afterward, it was used to investigate the effect of RNA quality on the blood gene expression profiles. All the sample characteristics on the basis of demography, blood cell counts and RNA quality were given in the Table 1.

**Table 1:** Sample information on the basis of demography, blood cell counts and RNA quality.

Variable	Men		Women		Total	
	Mean±SD	Study Range	Mean±SD	Study Range	Mean±SD	Study Range
***Age***	28.3±4.6	25–35	26.0±3.9	22–31	27.1±4.1	22–35
***BMI***	23.6±4.0	20.2–29.4	19.0±1.1	17.6–20.2	21.3±3.7	17.6–29.4
***Leukocyte (10∧9/l)***	6.8±2.9	3.6–10.6	5.6±0.5	5.0–6.1	6.2±2.0	3.6–10.6
***Lymphocyte%***	28.9±9.2	20.0–40.8	32.6±4.3	28.5**–**38.6	30.7±7.0	20.0**–**40.8
***Monocyte%***	7.0±0.9	6.8**–**7.9	5.7±1.1	4.7**–**7.2	6.4±1.2	4.7**–**7.9
***Neutrophil%***	61.2±9.7	48.4**–**69.4	59.8±5.0	52.4**–**62.8	60.5±7.2	48.4**–**69.4
***Erythrocyte (10∧12/l)***	5.5±0.4	4.9**–**5.9	4.3±0.3	4.1**–**4.7	4.9±0.7	4.1**–**5.9
***Hemoglobin (gm/dL)***	162.5±11.1	147**–**171	129.8±8.5	122**–**139	146.1±19.8	122**–**171
***Reticulocyte%***	0.5±0.2	0.3**–**0.7	0.4±0.4	0.2**–**1.0	0.5±0.3	0.2**–**1.0
***Platelet(10∧9/l)***	246.0±28.9	206**–**275	247.5±56.1	190**–**322	246.8±41.3	190**–**322
***RIN Value***	7.7±1.2	6.3**–**9.2	7.8±1.0	5.8**–**9.2	7.7±1.1	5.8**–**9.2

### Investigation of Variation in Blood Gene Expression Profiles

**Figure 3:**
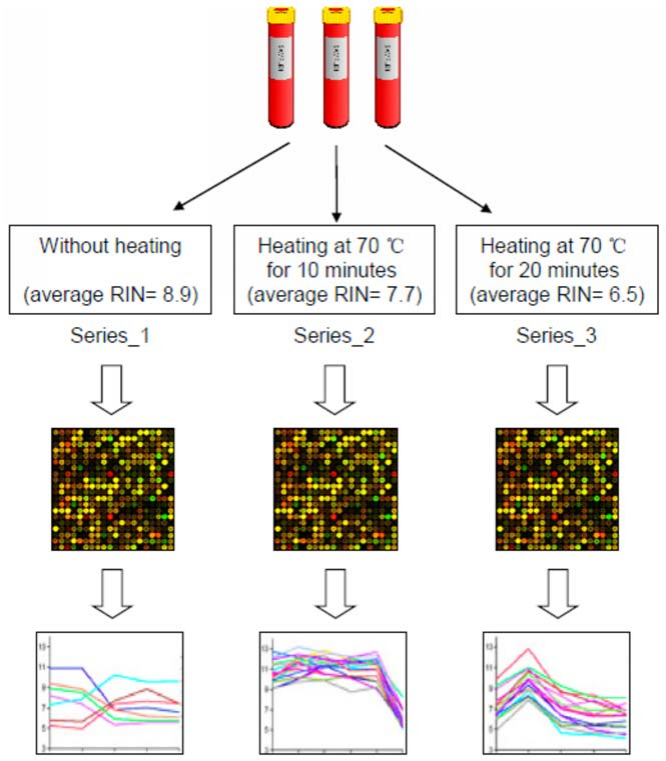
Illustrations of principle component analysis on the blood sample gene expression profiles. Principle component analysis was performed on gene expression profiles of 24 blood samples. Samples were displayed in three-dimensional space consisting of the Top-3 principal components which explain 55.4% of the variation of the whole dataset. Colours indicate blood samples collected from the same volunteers. Circles represent females and crosses represent male. The distances between samples reflect their approximate degree of transcriptional similarity. The individuals across three experimental series were in close proximity to one another, whereas females and males were separated.

Our blood gene expression dataset contained 9859 genes and 24 samples. The goal is to dissect the variation of thousands of gene expression profiles and characterise the underlying factors that contributed to the data variability. For this purpose, sophisticated data analysis methods were required. PCA is a widely used unsupervised linear technique for dimensionality reduction. The central idea behind PCA is to transform the original dataset consisting of a larger number of interrelated variables to a new set of uncorrelated principal components (PCs), while retaining as much as possible the variation present in the original data set [36]. Therefore, we used PCA to decompose the overall data variation into a set of PCs (Table S2). It was noteworthy that the Top-10 PCs explained 85% of the total variation, suggesting that the gene expression profiles might be affected by only few but significant factors. To explore and visualise the major sources of variation, samples were displayed in a 3-dimensional space consisting of the Top-3 PCs which explained 28.2%, 17.0% and 10.2% of the total variation, respectively. In the Figure 3, the spatial distance between the samples actually reflected their approximate degree of transcriptional similarity. Interestingly, the samples from each individual across experimental series were in close proximity to one another on the plane of PC1 and PC2, while male and female groups were separated in the direction of PC3. All of PCs transformed from original gene expression data were retained to avoid any information loss. Furthermore, a PCA extended method, called “*Eigen-R^2^*”, was used to precisely determine the proportion of variation related to the predefined biological and technological factors based on the PCA transformed data (Table 2).

**Table 2:** *Eigen-R^2^* analysis for dissecting data variation,variable-associated gene identification and functional annotation enrichment.

Variable	Proportion of Variation Explained by Variable (%)	N of Significant Genes(FDR<0.01)	Top Enriched Functional Annotation Term*
***Age***	8.3	196	blood coagulation, blood vessel development, stem cell maintenance, inflammatory response
***Gender***	9.2	105	iron ion binding, kinase binding, defense response, negative regulation of signal transduction
***BMI***	5.3	122	natural killer cell mediated cytotoxicity, hematopoietic cell lineage, phosphatase activity
***leukocyte***	12.6	557	leukocyte activation, mitochondrial membrane organization, homeostasis of number of cells, T cell receptor signaling pathway, immune response, cell activation, hemopoiesis, regulation of apoptosis
***lymphocyte%***	15.3	1146	
***monocyte%***	7.8	50	
***neutrophil%***	16.4	1560	
***erythrocyte***	8.6	299	negative regulation of apoptosis, iron ion binding, oxidation reduction, erythrocyte differentiation
***hemoglobin***	3.6	60	
***reticulocyte%***	3.7	7	
***platelet***	4.3	24	immune response, nucleotide binding,
***RIN value***	2.1	28	mRNA metabolic process, endoplasmic reticulum, chromatin organization

All the gene lists as well as the corresponding functional annotation enriched terms obtained via DAVID were provided in the Table S3.

### Variation Associated with RNA Quality and the Influence of RNA Degradation on Blood Gene Expression Profiles

It is widely believed that the highest quality RNA should be used for gene expression analyses. However, in some cases, such as human autopsy samples or paraffin embedded tissues, high quality RNA samples may not be available. Previous studies had investigated how the RNA quality might affect the gene expression profiles in the tissue samples [37], [38], remarkably little is know for the situation in blood. In this work, with a specific study design, we intended to explore the possible effect of sample quality on the blood gene expression profiles (Figure 1). The extracted total RNA from each individual was aliquoted into three tubes. The 1^st^ tube was kept intact, while the 2^nd^ and 3^rd^ tubes were heated at 70°C for 10 and 20 minutes, respectively. According to the RNAs preparation, microarray experiments were then performed in the sequential series: Series_1, Series_2 and Series_3. The average RIN value for Series_1, Series_2 and Series_3 were 8.9, 7.7 and 6.5, respectively. The distributions of RIN value between series were significantly different (*P*-value<0.001).

**Figure 4:**
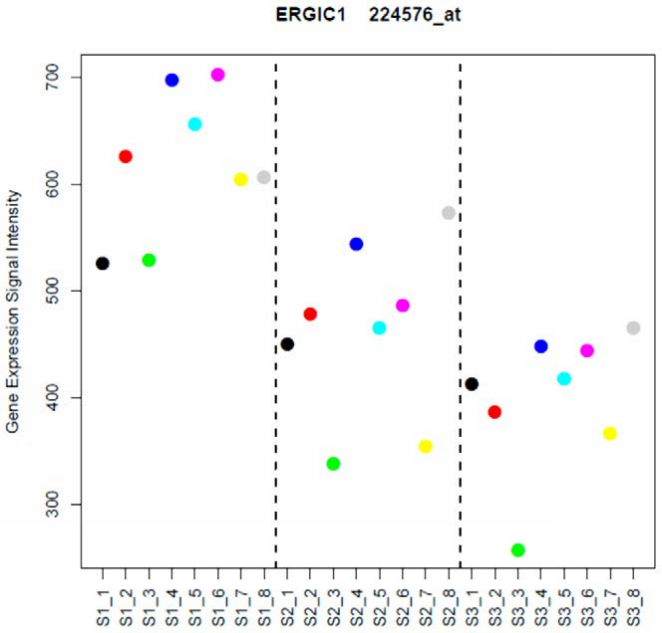
*ERGIC1* gene expression profiles across the series of arrays. In x-axis, the samples were arranged in accordance with the array Series 1–3 from the left to the right. Colours represent blood samples collected from the same volunteers. The y-axis indicates the gene expression signal intensity.

The RIN variable was shown to be associated with 2.1% of the total variation in blood gene expression data. The effect of RNA quality on the expression of individual genes was estimated through the gene-wise linear analysis at FDR <0.01. We identified 28 genes which the expression profiles were significantly associated with the RIN variable. The list of genes was submitted to the DAVID Bioinformatics Resource for functional enrichment analysis. The overview of variable-associated-gene list and enriched functional annotation terms were provided in the Table S3. The genes involved in “mRNA metabolic process” (*ZFP36L2, PTBP2, SFRS2IP, HNRNPC* and *RBM25*; *P*-value = 3.2E-3), “endoplasmic reticulum” (*CTSZ, CNIH4, SLMAP, ATF6B, CNPY3* and *ERGIC1*; *P*-value = 1.4E-2), as well as “chromatin organization” (*EPC1, HUWE1, MLL3* and *MPHOSPH8*; *P*-value = 2.5E-2) were significantly enriched. As an example, the expression profiles of *ERGIC1* (Endoplasmic Reticulum-Golgi Intermediate Compartment 1) were found highly variable between the series of arrays (Figure 4). Given the same cohort, the average expression intensity of *ERGIC1* dropped from 615 to 394 between Series_1 and Series_3, indicating the notable influence of sample RNA quality on the individual gene expression profiles. Recently, the technology advances such as RNA 6000 Nano Chips and Agilent 2100 Bioanalyzer had made it possible to access the samples' RNA quality beforehand. In case the quality of RNA was not satisfied, one would simply discard it. However, our results showed that this may not solve all the problems. Given the blood samples drawn from the same donors and high quality RNAs in Series_1 and Series_2 with the RIN values between 7.3 and 9.2, the expression intensity of *ERGIC1* were significantly different (*P*-value = 0.002). Hence, it could be also important to consider the distribution of RIN variable and make it equally distributed between the comparison groups.

### Variation Associated with Age

The aging effect might contribute to 8.3% of the total variation in blood gene expression profiles. A list of 196 genes was identified to be significantly associated with the age variable. Interestingly, several biological processes including “blood coagulation” (*CD40LG, CD59, TFPI, SERPING1* and *PF4*; *P*-value = 0.02), “blood vessel development” (*CEACAM1, EPAS1, IL8* and *TCF7L2*; *P*-value = 0.45), “stem cell maintenance” (*RIF1, TCL1A* and *TCF7L2*; *P*-value = 0.03*)* and “inflammatory response” (*C3AR1, TNFSF4, IL8, CD40LG, HRH4, RIPK2, SERPING1, IDO1* and *CXCL10*; *P*-value = 0.02) were significantly enriched. Given the fact that the blood vessel walls become thicker and tougher during the aging process, it was not unexpected that the angiogenesis-related genes were appeared in the list. On the other hand, the blood itself also changes with age in different aspects. The amount of bone marrow decreases with age, causing a decline in the formation of new blood cells. Therefore, recovery from bleeding episodes will be slowed. Age-related decline also occurs in white blood cells. Most of the white blood cells stay at the same levels, but certain white blood cells important to immunity (e.g. lymphocytes) decrease in their number and ability to resist inflammatory and infection.

We performed PCA and *Eigen-R^2^* analysis using the independent Karlovich *et al.* dataset. The age variable was found to be associated with 2.3% of the total variation. This value was in accordant with what had been reported by Karlovich *et al.*, however, somewhat lower compared to our result. We noticed some differences between two studies that might contribute to the discordance. The age range was smaller in our study (22–35 yrs) compared to the Karlovich *et al.* study (23–64 yrs). Actually, 14 out of 20 volunteers from Karlovich *et al.* dataset were above the 35 yrs old. Hence, it was not unexpected that our result could not be fully retrieved from those samples. Furthermore, Karlovich *et al.* explained that the broad age range in their study might have prevented the detection of the aging effect in blood [10]. Although similar observations had been reported previously [7], [11], the aging effect in blood transcriptome needs to be further specified with more samples and a broad range of ages.

### Variation Associated with Gender

The gender effect was found to be associated with 9.2% of the total variation in blood gene expression data. A total of 105 gender-associated genes were identified at FDR <0.01. It was intriguing that the Top-10 gender-associated genes were located on either the X or Y chromosomes (*XIST, RPS4Y1, EIF1AY, KDM5D, DDX3Y, CYorf15A, CYorf15B, USP9Y, UTY* and *PRKY*). The ectopic expression patterns of gene *XIST* (X Inactive Specific Transcript) was observed showing an average intensity of 3,000 in females and almost no expression in males. On the contrary, the Y chromosome linked gene *RPS4Y1* (Ribosomal Protein S4, Y-linked 1) was highly expressed in males with an average intensity of 1,800 but nearly no expression in females (Figure S1). In the Karlovich *et al.* dataset, gender effect was associated with 9.7% of the total variation, which was very similar to what was found in our dataset. Furthermore, the specific patterns of *XIST* and *RPS4Y1* were also retrieved. The ratio of *XIST* to *RPS4Y1* showed significantly higher in females compared to males (*P*-value = 1.6E-7), which might represent a useful gender-associated biomarker for the blood gene expression analyses in the future.

### Variation Associated with BMI

Given the distinct distribution of BMI between men and women, we included the gender variable to the *Eigen-R* ^2^ linear regression model for the estimation of variation. The BMI variable was found to be associated with 5.3% of the total variation. A total of 122 BMI-associated genes were identified at FDR <0.01. Of these genes, a cluster of 9 genes known to be related to “natural killer cell mediated cytotoxicity” were enriched (*TNFRSF10A*, *PRF1*, *KIR2DL5A*, *KIR2DS1*, *GZMB*, *KIR2DL1*, *KIR2DL3*, *SH2D1B* and *KIR3DL1*; *P*-value = 8.2E-6). Other genes involving in the “hematopoietic cell lineage” (*CD38, CD8B, IL7* and *CD5*; *P*-value = 0.03) and “phosphatase activity” (*ALPL, PTPRN2, INPP4B, PSPH* and *NT5E*; *P*-value = 0.05) were also enriched in the list.

### Variation Associated with White Blood Cells

**Figure 5:**
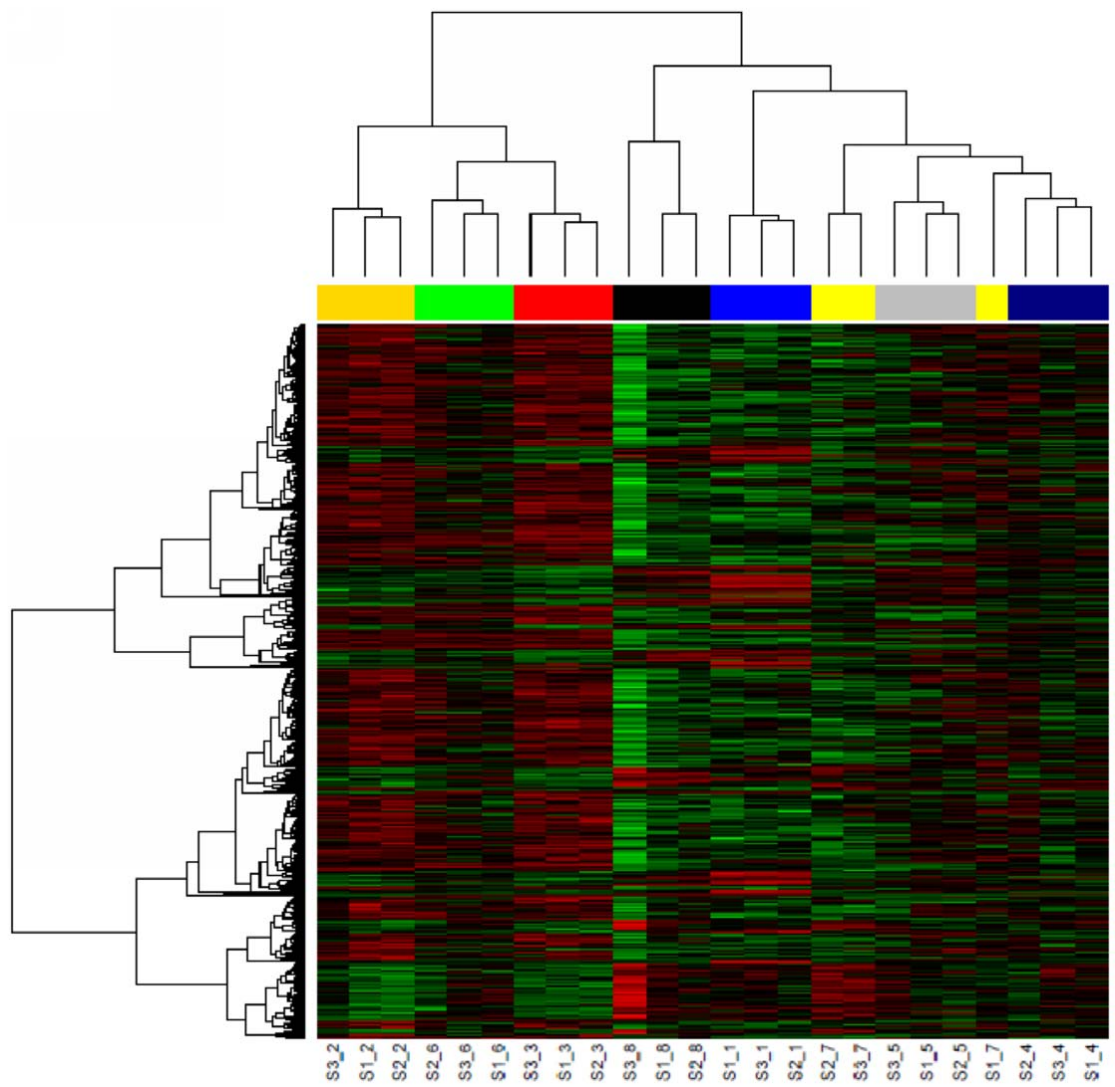
Hierarchical clustering of leukocytes-associated genes across samples exhibiting significant inter-individual differences. The expression profiles of 1893 leukocytes-associated genes across 24 samples were used for unsupervised hierarchical clustering. On the heat map, each row represents one gene and each column represents one patient. Red colour indicates gene over-expressed and green colour indicates gene low-expressed. Colours of bars associated with clustering of the columns designate samples from different donors. The leukocytes-associated genes perfectly cluster individual samples, suggesting the predominating effect of individual heterogeneities of leukocyte subsets on the blood gene expression profiles.

It was noteworthy that the white blood cell measures (“Leukocytes”, “Lymphocyte %”, “Monocyte %” and “Neutrophil %”) were associated with 7.8% to 16.4% of the total variation. Previously, white blood cells had been described as the most transcriptionally active of all cell types in blood and might present the most sensitive gene expression profiles in response to biological and technological factors [7], [11]. Taking the advantages of PCA and *Eigen-R* ^2^ method, our result demonstrated that the heterogeneity of white blood cell constituents indeed contributed to the most significant portion of the overall data variation. Via the gene-wise linear analysis at FDR<0.01, we identified 50, 557, 1146 and 1560 genes associated with “Monocyte %”, “Leukocytes”, “Lymphocyte %” and “Neutrophil %”, respectively. The white blood cell counts had been shown highly correlated, hence we combined the four gene sets together and resulted in a list of 1893 unique genes. As expected, numerous specific (e.g. “leukocyte activation”, *P*-value = 2.7E-7; “mitochondrial membrane organization”, *P*-value = 2.7E-5; “homeostasis of number of cells”, *P*-value = 8.1E-5; and “T cell receptor signalling pathway”, *P*-value = 1.1E-2) as well as more general biological processes (e.g. “immune response”, *P*-value = 4.2E-10; “cell activation”, *P*-value = 2.8E-8; “hemopoiesis”, *P*-value = 2.2E-5; and “regulation of apoptosis”, *P*-value = 1.8E-4) were significantly enriched among the list of leukocytes-associated genes. Hierarchical clustering of the leukocytes-associated genes resulted in perfect clustering of individual samples, demonstrating the predominating effect of individual heterogeneities of leukocyte subsets on the blood gene expression profiles (Figure 5).

### Variation Associated with Red Blood Cells

Given the different physiological conditions between men and women, the red blood cell counts were correlated with gender. Hence, we included the gender variable into the *Eigen-R* ^2^ linear regression model for the estimation of variation. The red blood measures (“reticulocyte%”, “erythrocyte” and “hemoglobin”) were found to be associated with 3.6% to 8.6% of the total variation. A total of 7, 60 and 299 genes were identified to be associated with “reticulocyte%”, “erythrocyte” and “hemoglobin”, respectively. The three lists were combined and resulted in a set of 306 unique genes. Interestingly, multiple relevant biological processes, such as: “negative regulation of apoptosis” (*HTATIP2, GNRH1, SOCS3, CLU, SNCA, PF4, PIM3, BCL2L1, STRADB, CSDA, MIF, PROK2, BAG1, HIPK3, BNIP3L, TGM2, MPO, BCL3, VNN1, BCL6, THBS1* and *F2R*; *P*-value = 4.1E-7), “iron ion binding” (*STEAP4, UTY, SNCA, EIF2AK1, CYP27A1, PGRMC1, HEBP1, HBZ, NDUFS8, MPO, SLC25A37, HBQ1, KDM6B* and *KDM5D*; *P*-value = 1.0E-3), “oxidation reduction” (*ACOX1, STEAP4, HTATIP2, DHRS13, UTY, SNCA, GMPR, SLC25A12, ADI1, AKR1C3, SNAI3, CYP27A1, PRDX6, BLVRB, NDUFS8, MPO, SMOX, ERO1L, KDM6B, NQO2* and *KDM5D*; *P*-value = 4.5E-3), as well as “erythrocyte differentiation” (*TAL1, HBZ, BCL6* and *KLF1*; *P*-value = 3.0E-2) were significantly enriched.

### Variation Associated with Platelets

Human blood platelets play critical roles in normal hemostatic processes and pathologic conditions such as inflammation, thrombosis, vascular remodelling, and wound repair. Although platelets are anucleate and lack nuclear DNA, they retain megakaryocyte-derived mRNAs [39]. Platelets contain rough endoplasmic reticulum and polyribosomes, and thereby they maintain the ability for protein biosynthesis from cytoplasmic mRNA [40]. Quiescent platelets generally display minimal translational activity, but newly formed platelets synthesize various α-granule and membrane glycoproteins [41]. In our dataset, the platelets variable was associated with 4.3% of the total variation. The gene-wise linear analysis identified 24 platelet-associated genes. Several genes involved in “immune response” (*DDX58, OAS3, RSAD2, IFI44L, OAS1, TREML1* and *IFI6*; *P*-value = 3.6E-5) as well as “nucleotide binding” (*OAS1, OAS3, DDX6, DDX58, NUAK1, CMPK2* and *RNF213*; *P*-value = 5.6E-3) were significantly enriched. Moreover, it was intriguing that *PEAR1* (platelet endothelial aggregation receptor 1) and *GP3A* (platelet glycoprotein III) were also found significantly associated, but with slightly higher FDRs at 0.02 and 0.03, respectively.

### Validation of the Microarray Data by Real-time PCR

Real-time PCR is generally considered the "gold-standard" assay for measuring gene expression and is often used to confirm findings from microarray data. A total of five genes (*C3AR1, XIST, LCK, OAS1* and *IFIT1*), which associated with age, gender and blood cell counts in microarray data, were selected for real-time PCR validation. The gene expression profiles determined by microarray and real-time PCR were found to be highly correlated (Table 3). Meanwhile, the real-time PCR based gene expression profiles were also significantly associated with the age, gender, lymphocyte%, reticulocyte% and platelet variables.

**Table 3:** Validation of microarray data by real-time PCR.

Gene	Associated variable	Correlations with variable		Correlations with microarray data	
		*ρ*	*P-*value	*ρ*	*P*-value
*C3AR1*	*age*	0.82	8.4 E-7	0.95	2.0 E-6
*XIST*	*gender*	0.87	4.2 E-8	0.75	2.0 E-5
*LCK*	*lymphocyte%*	0.6	0.002	0.73	7.2 E-5
*OAS1*	*reticulocyte%*	−0.65	0.001	0.87	2.5 E-6
*IFIT1*	*platelet*	−0.77	1.1 E-5	0.94	2.2 E-6

## Discussion

In the study described here, we intended to characterize the major sources of variation in the gene expression of human blood. We used the PCA and *Eigen-R* ^2^ method to dissect the overall variability of gene expression data and associate the major sources of variation with the predefined biological and technological factors. The results indicated that the predominating sources of the variation could be traced to the individual heterogeneity of the relative proportions of various blood cell types (leukocyte subsets and erythrocytes). The physiological factors (age, gender and BMI) were found to be associated with a significant proportion of variation in blood gene expression profiles. We further used a large independent dataset to perform the *in silico* validation. By applying the same statistical analyse, the similar gender effect as well as lessened aging effect was verified in the Karlovich *et al.* dataset. Through the specific design, we investigated the blood gene expression profiles from the same donors but with different levels of RNA quality. Although the proportion of variation associated to the RIN variable was mild, the significant impact of RNA quality on the expression of individual genes was observed.

Remarkably, the individual heterogeneities of blood cell constituents represent the most significant portion of the overall variation in blood gene expression. As a major defense and transport system, the blood interacts with virtually every organ and tissue in the human body, and thereby the gene expression responses of circulating white blood cells can potentially provide early warning of any abnormalities they discover. From this point of view, our results lend support to the clinical use of blood transcriptomic biomarkers for exposure, disease progression, diagnosis or prognosis. Given the significant effect of the relative proportions of different blood cell types, it is highly recommended to perform the CBC tests simultaneously and integrated the blood cell counts into the blood transcriptome study. For the donors with the blood cell counts falling outside normal ranges, they should be excluded. In some cases the size effects of biological or environmental factors are mite, the differences of gene expression profiles between comparison groups can be whelmed by the unconcerned individual heterogeneity and resulting in no differentially expressed genes was found. Then, it is possible to deconvolute blood gene expression profiles “*in silico*” if the measures of blood cell counts are available. This type of analysis can help to deduce cellular composition or cell-specific levels of gene expression using statistical methodologies [42]-[44]. Ultimately, increasing the sample size of comparison groups whenever possible is always helpful to neutralize the unconcerned individual heterogeneity. In the study design, physiological factors (age, gender and BMI) need to be well controlled and have them equally distributed between the comparison groups. The RNA quality of blood samples should be accessed before gene expression profiling. In case the quality of RNA not fit the predefined criteria, samples should be removed. It is also important to pay an attention to the global distribution of sample quality and make it well balanced between the comparison groups.

Finally, our study extends the limited information base currently available for the baseline variation of blood gene expression profiling in Chinese population. The clinical and transcriptomic data described in the study have been made freely available and should represent a useful resource for the design of future studies.

## Supporting Information

Figure S1 **The ectopic expression patterns of** ***XIST*** **and** ***RPS4Y1*** **in men and women.** In x-axis, the samples were arranged in accordance with the array Series 1–3 from the left to the right. Black and red dots represent blood samples collected from men and women, respectively. The y-axis indicates the gene expression signal intensity.
(TIF)

Table S1 **Primers of selected genes for real-time PCR.**
(DOC)

Table S2 **The proportion of total variation explained by each principle component.**
(DOC)

Table S3 **The overview of variable-associated-gene lists and enriched functional annotation terms.**
(XLS)

## References

[1] Chaussabel D, Pascual V, Banchereau J (2010). Assessing the human immune system through blood transcriptomics.. BMC Biol.

[2] Staratschek-Jox A, Classen S, Gaarz A, Debey-Pascher S, Schultze JL (2009). Blood-based transcriptomics: leukemias and beyond.. Expert Rev Mol Diagn.

[3] Fan H, Hegde PS (2005). The transcriptome in blood: challenges and solutions for robust expression profiling.. Curr Mol Med.

[4] Radich JP, Mao M, Stepaniants S, Biery M, Castle J (2004). Individual-specific variation of gene expression in peripheral blood leukocytes.. Genomics.

[5] Min JL, Barrett A, Watts T, Pettersson FH, Lockstone HE (2010). Variability of gene expression profiles in human blood and lymphoblastoid cell lines.. BMC Genomics.

[6] Palmer C, Diehn M, Alizadeh AA, Brown PO (2006). Cell-type specific gene expression profiles of leukocytes in human peripheral blood.. BMC Genomics.

[7] Whitney AR, Diehn M, Popper SJ, Alizadeh AA, Boldrick JC (2003). Individuality and variation in gene expression patterns in human blood.. Proc Natl Acad Sci U S A.

[8] Tang Y, Lu A, Ran R, Aronow BJ, Schorry EK (2004). Human blood genomics: distinct profiles for gender, age and neurofibromatosis type 1.. Brain Res Mol Brain Res.

[9] Dumeaux V, Olsen KS, Nuel G, Paulssen RH, Borresen-Dale AL (2010). Deciphering normal blood gene expression variation–The NOWAC postgenome study.. PLoS Genet.

[10] Karlovich C, Duchateau-Nguyen G, Johnson A, McLoughlin P, Navarro M (2009). A longitudinal study of gene expression in healthy individuals.. BMC Med Genomics.

[11] Eady JJ (2005). Variation in gene expression profiles of peripheral blood mononuclear cells from healthy volunteers.. Physiological Genomics.

[12] Liu J, Walter E, Stenger D, Thach D (2006). Effects of globin mRNA reduction methods on gene expression profiles from whole blood.. J Mol Diagn.

[13] Debey S, Zander T, Brors B, Popov A, Eils R (2006). A highly standardized, robust, and cost-effective method for genome-wide transcriptome analysis of peripheral blood applicable to large-scale clinical trials.. Genomics.

[14] Vartanian K, Slottke R, Johnstone T, Casale A, Planck SR (2009). Gene expression profiling of whole blood: comparison of target preparation methods for accurate and reproducible microarray analysis.. BMC Genomics.

[15] Thach DC, Lin B, Walter E, Kruzelock R, Rowley RK (2003). Assessment of two methods for handling blood in collection tubes with RNA stabilizing agent for surveillance of gene expression profiles with high density microarrays.. J Immunol Methods.

[16] Debey S, Schoenbeck U, Hellmich M, Gathof BS, Pillai R (2004). Comparison of different isolation techniques prior gene expression profiling of blood derived cells: impact on physiological responses, on overall expression and the role of different cell types.. Pharmacogenomics J.

[17] Kim SJ, Dix DJ, Thompson KE, Murrell RN, Schmid JE (2007). Effects of storage, RNA extraction, genechip type, and donor sex on gene expression profiling of human whole blood.. Clin Chem.

[18] Wang J, Robinson JF, Khan HM, Carter DE, McKinney J (2004). Optimizing RNA extraction yield from whole blood for microarray gene expression analysis.. Clin Biochem.

[19] Li L, Roden J, Shapiro BE, Wold BJ, Bhatia S (2005). Reproducibility, fidelity, and discriminant validity of mRNA amplification for microarray analysis from primary hematopoietic cells.. J Mol Diagn.

[20] Rainen L, Oelmueller U, Jurgensen S, Wyrich R, Ballas C (2002). Stabilization of mRNA expression in whole blood samples.. Clin Chem.

[21] Ihaka R, Gentleman R (1996). R: A Language for Data Analysis and Graphics.. Journal of Computational and Graphical Statistics.

[22] Gentleman RC, Carey VJ, Bates DM, Bolstad B, Dettling M (2004). Bioconductor: open software development for computational biology and bioinformatics.. Genome Biol.

[23] Bolstad BM, Irizarry RA, Astrand M, Speed TP (2003). A comparison of normalization methods for high density oligonucleotide array data based on variance and bias.. Bioinformatics.

[24] Irizarry RA, Hobbs B, Collin F, Beazer-Barclay YD, Antonellis KJ (2003). Exploration, normalization, and summaries of high density oligonucleotide array probe level data.. Biostatistics.

[25] Irizarry RA, Bolstad BM, Collin F, Cope LM, Hobbs B (2003). Summaries of Affymetrix GeneChip probe level data.. Nucleic Acids Res.

[26] Wilson CL, Miller CJ (2005). Simpleaffy: a BioConductor package for Affymetrix Quality Control and data analysis.. Bioinformatics.

[27] Chen LS, Storey JD (2008). Eigen-R2 for dissecting variation in high-dimensional studies.. Bioinformatics.

[28] Schroeder LD, Sjoquist DL, Stephan PE (1986). Understanding regression analysis: an introductory guide..

[29] Smyth GK, Gentleman R, Carey V, Dudoit S, Irizarry R, Huber W (2005). Limma: linear models for microarray data.. Bioinformatics and Computational Biology Solutions using R and Bioconductor.

[30] Benjamini Y, Hochberg Y (1995). Controlling the False Discovery Rate: A Practical and Powerful Approach to Multiple Testing.. Journal of the Royal Statistical Society.

[31] Huang Da W, Sherman BT, Lempicki RA (2009). Systematic and integrative analysis of large gene lists using DAVID bioinformatics resources.. Nat Protoc.

[32] Brazma A, Hingamp P, Quackenbush J, Sherlock G, Spellman P (2001). Minimum information about a microarray experiment (MIAME)-toward standards for microarray data.. Nat Genet.

[33] Parkinson H, Kapushesky M, Shojatalab M, Abeygunawardena N, Coulson R (2007). ArrayExpress–a public database of microarray experiments and gene expression profiles.. Nucleic Acids Res.

[34] Barrett T, Edgar R (2006). Gene expression omnibus: microarray data storage, submission, retrieval, and analysis.. Methods Enzymol.

[35] Schroeder A, Mueller O, Stocker S, Salowsky R, Leiber M (2006). The RIN: an RNA integrity number for assigning integrity values to RNA measurements.. BMC Mol Biol.

[36] Jolliffe IT (2002). Principal Component Analysis..

[37] Luzzi V, Mahadevappa M, Raja R, Warrington JA, Watson MA (2003). Accurate and reproducible gene expression profiles from laser capture microdissection, transcript amplification, and high density oligonucleotide microarray analysis.. J Mol Diagn.

[38] Schoor O, Weinschenk T, Hennenlotter J, Corvin S, Stenzl A (2003). Moderate degradation does not preclude microarray analysis of small amounts of RNA.. Biotechniques.

[39] Wissler C (1905). The Spearman Correlation Formula.. Science.

[40] Kieffer N, Guichard J, Farcet JP, Vainchenker W, Breton-Gorius J (1987). Biosynthesis of major platelet proteins in human blood platelets.. Eur J Biochem.

[41] Gnatenko DV, Dunn JJ, Schwedes J, Bahou WF (2009). Transcript profiling of human platelets using microarray and serial analysis of gene expression (SAGE).. Methods Mol Biol.

[42] Shen-Orr SS, Tibshirani R, Khatri P, Bodian DL, Staedtler F (2010). Cell type-specific gene expression differences in complex tissues.. Nat Methods.

[43] Lu P, Nakorchevskiy A, Marcotte EM (2003). Expression deconvolution: a reinterpretation of DNA microarray data reveals dynamic changes in cell populations.. Proc Natl Acad Sci U S A.

[44] Abbas AR, Wolslegel K, Seshasayee D, Modrusan Z, Clark HF (2009). Deconvolution of blood microarray data identifies cellular activation patterns in systemic lupus erythematosus.. PLoS One.

