# Supplementary figures and images for: Investigation of Variation in Gene Expression Profiling of Human Blood by Extended Principle Component Analysis
Source: PLoS One. 2011 Oct 27;6(10):e26905. doi: 10.1371/journal.pone.0026905 (PMC3203156; doi:10.1371/journal.pone.0026905)

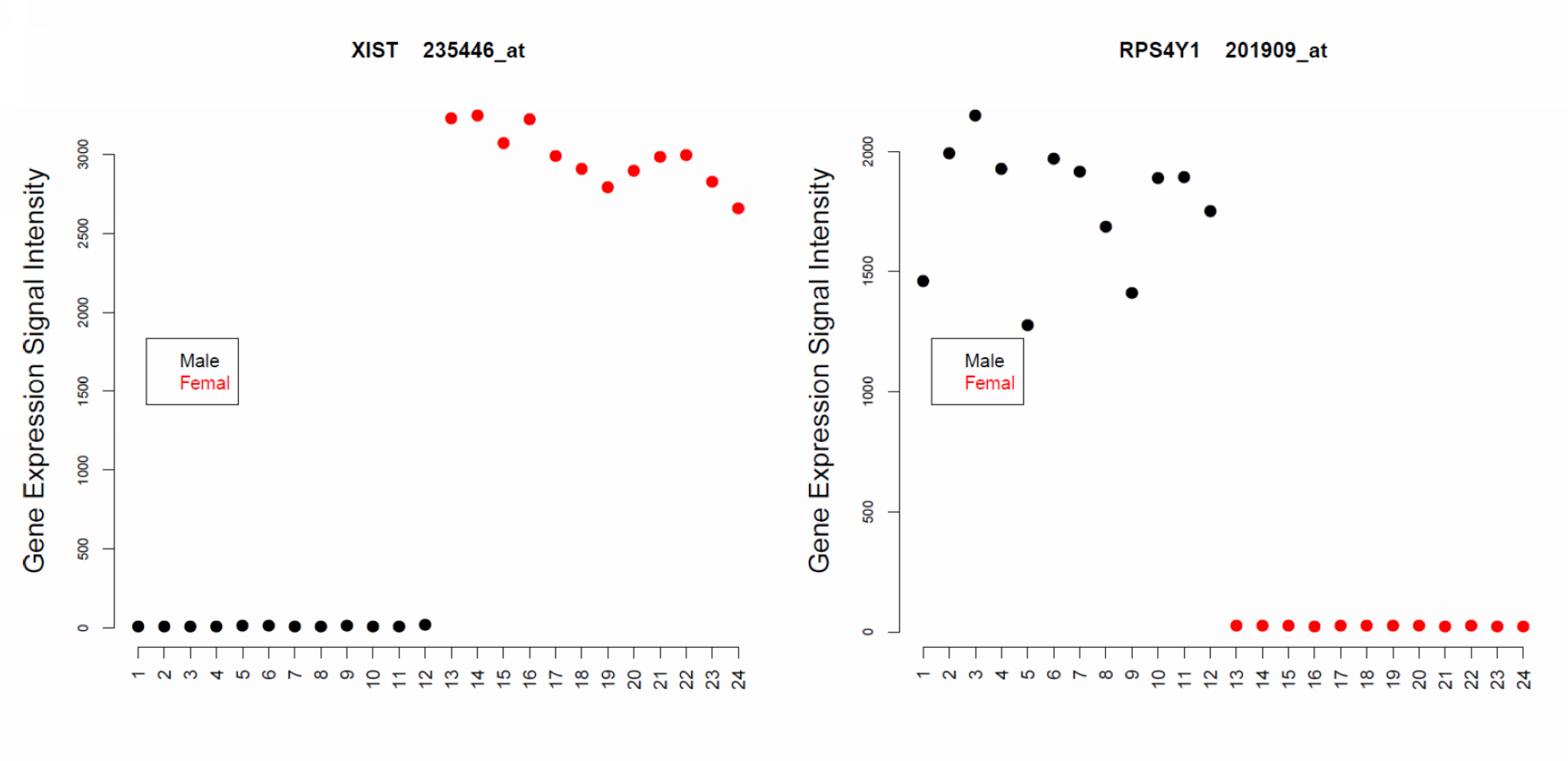

Supplement: Figure S1 — The ectopic expression patterns of XIST and RPS4Y1 in men and women. In x-axis, the samples were arranged in accordance with the array Series 1–3 from the left to the right. Black and red dots represent blood samples collected from men and women, respectively. The y-axis indicates the gene expression signal intensity. (TIF) [file pone.0026905.s001.tif]
