# Supplementary material for: Investigation of Variation in Gene Expression Profiling of Human Blood by Extended Principle Component Analysis
Source: PLoS One. 2011 Oct 27;6(10):e26905. doi: 10.1371/journal.pone.0026905 (PMC3203156; doi:10.1371/journal.pone.0026905)
Supplement: Table S1 — Primers of selected genes for real-time PCR. (DOC) [file pone.0026905.s002.doc]

| Gene | Forward | Reverse |
| --- | --- | --- |
| *C3AR1* | CCTGCTGATGTGGTCTCACCTA | CCTTGTGGTAGCTCAGACTCGT |
| *XIST* | GTAGGTGTGCTGATAACCAAGGC | GGGAAAGGAAGATTGAGGGTGG |
| *LCK* | AACACTCACGGCTCCTTCCTCA | GTAGAAGCCACCGTTGTCCAGA |
| *OAS1* | AGGAAAGGTGCTTCCGAGGTAG | GGACTGAGGAAGACAACCAGGT |
| *IFIT1* | GCCTTGCTGAAGTGTGGAGGAA | ATCCAGGCGATAGGCAGAGATC |
