# Supplementary material for: Investigation of Variation in Gene Expression Profiling of Human Blood by Extended Principle Component Analysis
Source: PLoS One. 2011 Oct 27;6(10):e26905. doi: 10.1371/journal.pone.0026905 (PMC3203156; doi:10.1371/journal.pone.0026905)
Supplement: Table S2 — The proportion of total variation explained by each principle component. (DOC) [file pone.0026905.s003.doc]

| **Principle Component** | **PC1** | **PC2** | **PC3** | **PC4** | **PC5** | **PC6** | **PC7** | **PC8** | **PC9** | **PC10** | **PC11** | **PC12** |
| --- | --- | --- | --- | --- | --- | --- | --- | --- | --- | --- | --- | --- |
| **Proportion of variation explained (%)** | 28.2 | 17.0 | 10.2 | 7.4 | 5.7 | 4.8 | 4.1 | 3.8 | 2.9 | 1.7 | 1.4 | 1.3 |
| **Principle Component** | **PC13** | **PC14** | **PC15** | **PC16** | **PC17** | **PC18** | **PC19** | **PC20** | **PC21** | **PC22** | **PC23** | **PC24** |
| **Proportion of variation explained (%)** | 1.3 | 1.1 | 1.1 | 1.1 | 1.1 | 1.0 | 1.0 | 0.9 | 0.9 | 0.9 | 0.9 | 0.0 |
